# The HIV-1 Integrase Mutations Y143C/R Are an Alternative Pathway for Resistance to Raltegravir and Impact the Enzyme Functions

**DOI:** 10.1371/journal.pone.0010311

**Published:** 2010-04-26

**Authors:** Sandrine Reigadas, Guerric Anies, Bernard Masquelier, Christina Calmels, Lieven J. Stuyver, Vincent Parissi, Herve Fleury, Marie-Line Andreola

**Affiliations:** 1 Laboratoire de Virologie, CHU de Bordeaux, EA 2968, Université Victor Segalen, Bordeaux, France; 2 UMR 5234, CNRS, Université Victor Segalen, Bordeaux, France; 3 Virco BVBA, Mechelen, Belgium; University of California San Francisco, United States of America

## Abstract

Resistance to HIV-1 integrase (IN) inhibitor raltegravir (RAL), is encoded by mutations in the IN region of the *pol* gene. The emergence of the N155H mutation was replaced by a pattern including the Y143R/C/H mutations in three patients with anti-HIV treatment failure. Cloning analysis of the IN gene showed an independent selection of the mutations at loci 155 and 143. Characterization of the phenotypic evolution showed that the switch from N155H to Y143C/R was linked to an increase in resistance to RAL. Wild-type (WT) IN and IN with mutations Y143C or Y143R were assayed *in vitro* in 3′end-processing, strand transfer and concerted integration assays. Activities of mutants were moderately impaired for 3′end-processing and severely affected for strand transfer. Concerted integration assay demonstrated a decrease in mutant activities using an uncleaved substrate. With 3′end-processing assay, IC_50_ were 0.4 µM, 0.9 µM (FC = 2.25) and 1.2 µM (FC = 3) for WT, IN Y143C and IN Y143R, respectively. An FC of 2 was observed only for IN Y143R in the strand transfer assay. In concerted integration, integrases were less sensitive to RAL than in ST or 3′P but mutants were more resistant to RAL than WT.

## Introduction

Retroviral integration, which is an essential step for viral replication, is performed by viral integrase (IN). HIV-1 IN is a 32 kDa protein with three different domains [1]. The catalytic core contains the catalytic triad DDE which is the signature of enzymes belonging to the polynucleotidyl transferase family [2]. The N-terminal domain contains a pair of conserved His and Cys residues that coordinate a single zinc atom. When deleted, the C terminal part leads to the loss of DNA binding capacity. The enzyme catalyses two steps: cleavage of the two 3′-end nucleotides of each LTR (3′-end processing), thereby producing CpA 3′hydroxyl ends; and transesterification leading to the integration of both viral ends in the cellular DNA (strand transfer reaction). As this step is crucial for viral replication, numerous studies have been conducted to design HIV-1 integrase inhibitors (INI) that block the integration of viral double-stranded DNA into the host cell's chromosomal DNA [3]. Two classes of inhibitors, interfering either with the 3′ processing of the viral DNA long terminal repeats [4], [5] or with the strand transfer of viral DNA into the host genome [6], have been described. Raltegravir (RAL) is an integrase strand transfer inhibitor which has shown antiretroviral activity in antiretroviral-naïve [7] and –experienced patients [8], [9], and is to date the only INI approved for therapeutic use.

Resistance to RAL has been described *in vitro* and *in vivo*. The most frequent primary RAL resistance mutations emerging *in vivo* at virological failure (VF) in the IN gene are Q148H/R/K, N155H, and to a lesser extent Y143C/H/R [10]. Numerous other mutations considered as secondary RAL resistance mutations have also been described [11]. *In vitro*, several mutations have been introduced into the IN gene and activities of mutants have been determined (T66I, L74M, E92Q, F121Y, Q148K, S153Y, N155H) [12]. In general, all resistant enzymes were at least partially impaired for strand transfer function. In some cases, 3′end processing was also impaired. In general, the mutants showed an inverse correlation between resistance and catalytic activity [12]. The Q148H mutation, which leads to a decreased activity of IN, confers a higher level of resistance to RAL than G140S or N155H. Q148H is rescued by the G140S mutation [13]. Nevertheless, both activities of 3′end processing and strand transfer are highly impaired in the double mutant.

In a previous work, we reported how patients who had failed to respond to therapies under RAL-containing regimens presented the N155H mutation, which was then replaced over time by the Y143C/H/R mutations [14]. We describe here the genetic pathways and the dynamics of emergence of the Y143C/R mutations in HIV-1 integrase, and the impact of these mutations on the enzymatic functions of the integrase in the presence or absence of RAL.

## Results

### Baseline characteristics and follow-up

Three patients with virological failure on RAL and with selection of mutations at IN position 143 were included in the study. The baseline characteristics of three patients and the co-prescribed drugs within the optimized regimen are described in Table 1. The patients had experienced multiple virological failures on ART and had few treatment options left, as shown by their low GSS. The three HIV-1 strains clustered with HIV-1 subtype B.

**Table 1:** Baseline characteristics of three patients failing on raltegravir-containing regimens.

Patient	VL	CD4	Co-prescribed drugs	RT mutations	PR mutations	GSS
1	6.7	16	ETR/DRV/r	41L 44D 67N 69D/N 74I 75T 101E 179F 181C 184V 190S 210W 215Y	10F 15V 20R 32I 33F 36I 50V 54A 62V 63P 71V 84V 85V 89V 90M	0
2	6.4	36	ETR/DRV/r/ENF	41L 44D 67N 74V 103N 184V 210W 215Y	10F/V 15V 20R 33F 46I 62V 63P 71T 73S 76V 84V 89V 90M	1
3	5.6	22	TDF/FTC/ETR/DRV/r	41L 44D 67N 70R 181C 184V 190A 210W 215Y	10V 15V 20R 32I 33F 46I 54V 58E 63P 82A 84V 90M	0

Abbreviations: VL: Viral load (Plasma HIV-1 RNA), log_10_copies/ml; CD4: CD4 (+) cell count (cells/µl); GSS: Genotypic sensitivity score;

TDF: tenofovir diphospho fumarate; ETR: etravirine; DRV/r: darunavir boosted with ritonavir; ENF: enfuvirtide; FTC: emtricitabine.

**Figure 1:**
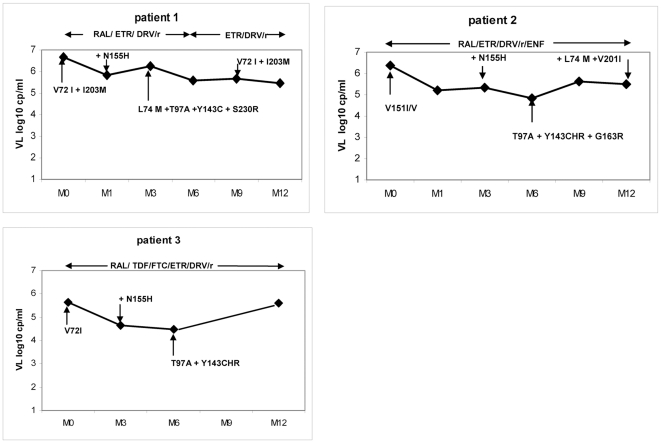
Virological evolution in three patients with virological failure on Raltegravir-containing therapy. Time of follow-up is expressed in months (M0 to M12). Baseline integrase polymorphisms appear at month 0. Additional selected mutations preceded by (+), or switches to different genotypic patterns, are indicated in the follow-up. Abbreviations: VL: Viral load (Plasma HIV-1 RNA); RAL: raltegravir; TDF: tenofovir diphospho fumarate; ETR: etravirine; DRV/r: darunavir boosted with ritonavir; ENF: enfuvirtide; FTC: emtricitabine.

The evolution of plasma HIV-1 RNA on RAL in the three patients is shown in Figure 1. All three patients had a limited decrease in plasma HIV-1 RNA, without a decrease below the detection threshold of 50 copies/mL. The CD4 (+) cell evolution showed no (patients 1 and 2) or a weak (patient 3) immunological response to RAL.

### Selection of INI resistance mutations

The selection of INI resistance mutations on RAL-containing therapy is shown in Table 2. The IN sequences were first determined at baseline (D0) before prescription of RAL. Several polymorphic mutations previously related to INI resistance were present at D0: V72I was present in patients 1 and 3, V151I in patient 2, and I203M in patient 1. A particular evolution of genotypic pattern could be characterized when we studied the selection of INI resistance mutations on RAL-containing regimens. In all three patients, the initial selection of the N155H mutation was followed by its disappearance and replacement by a pattern comprising the Y143H/R/C mutations with other mutations (T97A in 3 patients, L74M in 2 patients and G163R and S230R in one patient each); RAL was stopped between months 6 and 12 in patient 1, with disappearance of the selected mutations.

**Table 2:** Genotypic evolution of integrase and phenotypic sensitivity to raltegravir and elvitegravir in 3 patients failing on raltegravir-containing regimens.

Patient N°	Time on RAL	ART	Viral load (log_10_copies/ml)	CD4 (cells/mm^3^)	Integrase mutations	RAL FC	EVG FC
1	D0	RAL/ETR/DRV/r	6.7	16	V72I I203M	0.89	0.89
	M1	RAL/ETR/DRV/r	5.8	20	V72I N155N/H I203M	1.07	2.1
	M3	RAL/ETR/DRV/r	6.3	4	V72I L74L/M T97A/T Y143C I203M	5.87	1.56
	M6	RAL/ETR/DRV/r	5.6	10	V72I L74L/M T97A/T Y143C/Y I203M	n.d.	n.d.
	M9	ETR/DRV/r*	5.7	10	V72I I203M	0.98	0.83
	M12	ETR/DRV/r*	5.5	7	V72I I203M	0.76	1.43
2	D0	RAL/ETR/DRV/r/ENF	6.4	36	V151I/V	0.76	0.94
	M1	RAL/ETR/DRV/r/ENF	5.2	77	n.d.	n.d.	n.d.
	M3	RAL/ETR/DRV/r/ENF	5.4	67	N155N/H	1.72	6.05
	M6	RAL/ETR/DRV/r/ENF	4.8	36	T97A/T G163G/R Y143Y/C/H/R	28.52	6.23
	M9	RAL/ETR/DRV/r/ENF	5.6	31	T97A/T G163G/R Y143Y/C/H/R	21.13	2.68
	M12	RAL/ETR/DRV/r/ENF	5.5	12	L74L/M T97A/T G163G/R Y143Y/C/H/R V201I/V	n.d.	n.d.
3	D0	RAL/TDF/FTC/ETR/DRV/r	5.6	22	V72I	0.91	0.60
	M1	RAL/TDF/FTC/ETR/DRV/r	4.7	48	V72I	n.d.	n.d.
	M3	RAL/TDF/FTC/ETR/DRV/r	4.5	88	V72I N155N/H	n.d.	n.d.
	M6	RAL/TDF/FTC/ETR/DRV/r	5.6	103	V72I T97A/T Y143C/H/R/Y	51.95	3
	M12	RAL/TDF/FTC/ETR/DRV/r	6.2	69	V72I T97A/T Y143C/H/R/Y	n.d.	9.57

Abbreviations: Time on RAL: duration of raltegravir–based therapy (months); *: raltegravir has been removed from therapy after month 6 for patient 1; RAL FC: raltegravir sensitivity fold-change; EVG FC: elvitegravir sensitivity fold-change; TDF: tenofovir diphospho fumarate; ETR: etravirine; DRV/r: darunavir boosted with ritonavir; ENF: enfuvirtide; FTC: emtricitabine; n.d.: not determined.

### Phenotypic sensitivity to RAL and EVG

The RAL and EVG FC are shown in Table 2. Phenotypic resistance is expressed in terms of the FC in IC_50_ to each drug for the virus in question compared to a wild-type reference virus. The two samples with the N155H had low RAL FC (1.07 and 1.72) and low EVG FC (2. and 6.05). In samples with the Y143C/H/R mutations, the median baseline RAL FC was 24.83 (range 5.87–51.95) and the median baseline EVG FC was 3 (range 1.56–9.57).

### Clonal analysis of HIV-1 integrase

In order to study the dynamics of replacement and the linkage between mutations in patients presenting a switch from the N155H to the Y143C/H/R pattern, we cloned and sequenced the viral populations from the three patients at baseline RAL and during follow-up. In all three patients, the replacement over time of the N155H pattern by the Y143R/C pattern was confirmed by clonal sequences (Table S1). In patient 1, N155H was present in 39% of clones at M1 and in 9% of clones at M3, whereas Y143C was absent at M1 and present in 91% of clones at M3. In patient 2, the percentages of clones with the N155H/R mutations and with the Y143C mutation were respectively 84% and 9% at M3, and 0% and 66% at M6. In patient 3, the N155H mutation was detected only at M3 and in 72% of clones, whereas Y143R was present only at M6 and in 47% of clones. The mutations at residues 143 and 155 were never present on the same clonal sequence in any of the three patients. Phylogenetic analyses comprising all clonal sequences for each patient suggested an independent selection of the mutations at loci 143 and 155 (data not shown). Clonal analysis also showed that the T97A and G163R mutations could be present in association with Y143R but could also be selected in the absence of Y143R (in patients 2 and 3).

### Comparison of catalytic activities of wild-type and mutant IN

**Figure 2:**
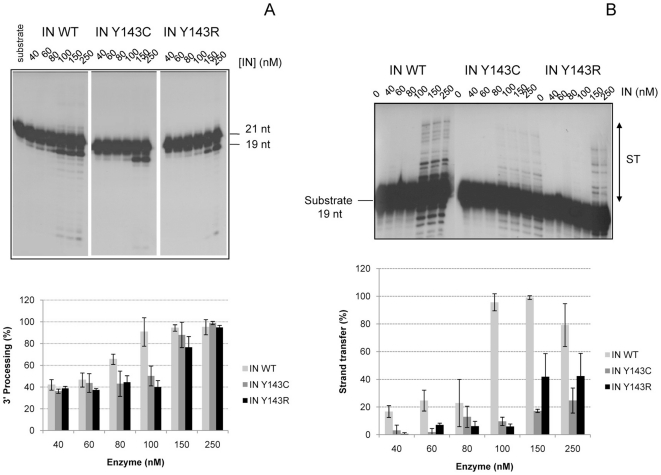
Comparison of IN WT and IN Y143C/R activities *in vitro.* **2A: 3′-Processing activity of IN WT and IN Y143C/R mutants.** Different concentrations of wild-type (IN WT) or mutated HIV-1 integrases were incubated for 1 h at 37°C with the 3′end processing substrate as indicated in method. Samples were analyzed by electrophoresis on 16% acrylamide gels/ 7 M urea in TBE. Top: A typical electrophoresis is shown. Bottom: Quantification of 3′-end processing activity of IN WT and mutants is shown. Data are the average of at least three different experiments. **2B: Strand transfer activity of IN WT and IN Y143C/R mutants.** Increasing amounts of WT and mutated IN were incubated with the strand transfer substrate for 1 h at 37°C. A typical experiment is shown (top). The quantification of activities of IN WT and mutants is shown at bottom. Data are the average of at least three different experiments.

Mutations Y143C and Y143R were introduced into the IN gene and recombinant enzymes were expressed and purified according to standard procedures used in the laboratory [15]. The activities of 3′-end processing, strand transfer (ST) and concerted integration were compared to the wild-type one. 3′end-processing activities were moderately impaired compared to the wild-type enzyme, since 80% of the activity of the wild type is still present (Figure 2A). On the other hand, the strand transfer activities of the mutant enzymes were severely impaired, since only 30% (IN Y143C) and 50% (IN Y143R) of the activity of the wild type was measured at a concentration of 250 nM of enzyme (Figure 2B).

**Figure 3:**
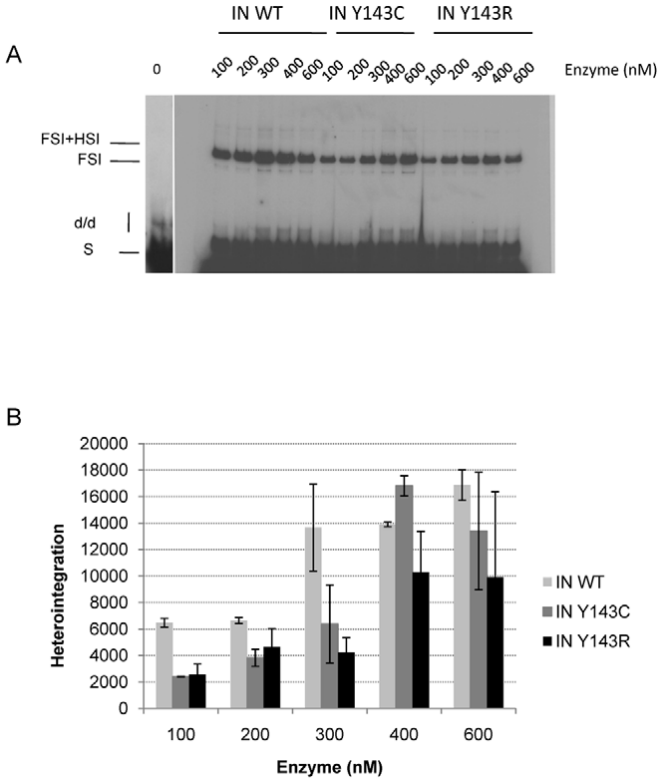
Concerted integration activity of IN WT and IN Y143C/R mutants. Increasing amounts of IN WT or mutants IN Y143C or Y143R were incubated in presence of donor and acceptor DNA. Reaction was then processed as described in material and methods. An example of products obtained in a typical experiment is illustrated in fig 3A. The position and the structure of the different products half-site (HSI), full-site (FSI) and donor/donor integration (d/d) are shown. Figure 3B corresponds to the quantification of several independent experiments. Heterointegration corresponds to densitometry of the FSI and FSI+HIS bands (see products indicated in Figure 3A).

The activity of the mutants was also compared using concerted integration assay and a blunt-ended substrate as described in [16], [17]. Integration in these conditions necessitates processing then strand transfer activity of the two ends of a donor substrate mimicking the 5′ and 3′ends of viral LTRs. Products of integration (full-site and half-site, FSI and HSI respectively) were in agreement with products expected from previous work [16], [18]. In particular for enzymes concentrations below 400 nM, the activity of the mutants was decreased in comparison to that of the wild-type enzyme, with a slightly lower activity for mutation Y143R (Figure 3).

### Comparison of sensitivities of wild-type and mutant IN to RAL

**Figure 4:**
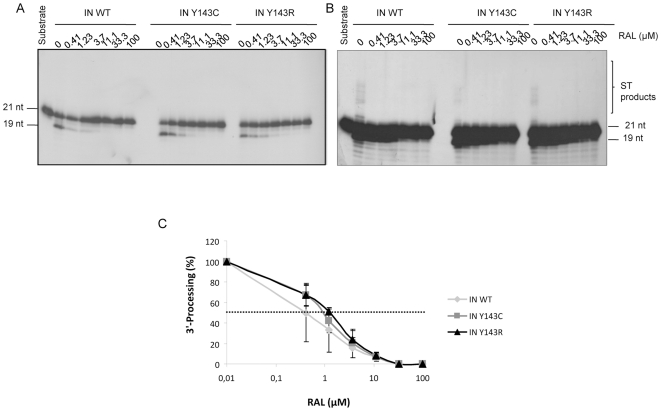
Sensitivity to RAL of IN WT and IN Y143C/R mutants in a 3′-processing assay. IN (150 nM) was incubated in the presence of increasing amounts of RAL. Then activity was assayed in the presence of a 3′end processing substrate. A typical analysis is shown in Figure 4A (2-hour exposure) and 4B (overnight exposure). Inhibition curves are shown in Figure 4C and are the result of at least three independent experiments.

The mutants were first tested for their sensitivity to RAL in a 3′end processing assay. All three enzymes, IN WT, IN Y143C and IN Y143R, were sensitive to RAL (Figure 4A) with an IC_50_ for 3′-end processing of 0.4 µM, 0.9 µM (FC = 2.25) and 1.2 µM respectively (FC = 3). In the same 3′end processing assay, strand transfer activity resulting from the 3′end processing was also observed. The strand transfer of the three enzymes was inhibited (Figure 4B). ST was more sensitive to RAL than processing, thus showing the specificity of RAL for ST. Complete inhibition of ST was reached with 0.4 µM of drug while complete inhibition of 3′end processing necessitated a 10-fold higher concentration of drug when starting from an uncleaved substrate (see Figure 4B, longer exposure of Figure 4A).

**Figure 5:**
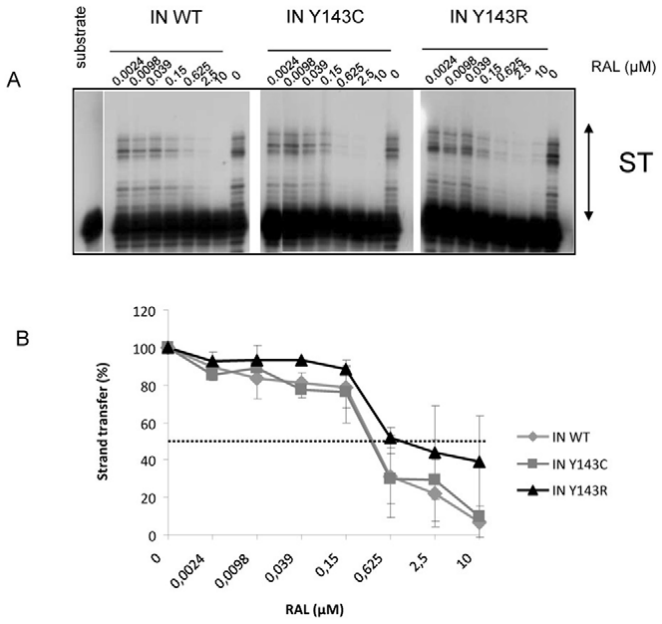
Sensitivity to RAL of IN WT and IN Y143C/R mutants in a strand transfer assay. IN (150 nM) was incubated in the presence of increasing amounts of RAL. Then activity was assayed in the presence of a strand transfer substrate. A typical gel is shown if Figure 5. Inhibition curves are shown in Figure 5B and are the result of at least three independent experiments.

IC_50_ for strand transfer activity were also determined in an *in vitro* dose response assay with precleaved substrate (Figure 5). All three enzymes were sensitive to RAL inhibition. IC_50_ was around 0.3 µM for IN WT and IN Y143C (Figure 5). On the contrary, the Y143R mutation conferred a decreased sensitivity to RAL (FC = 2). A Student test that was used to derive P values showed there are not statistical differences between the IN WT and Y143C, and Y143R.

**Figure 6:**
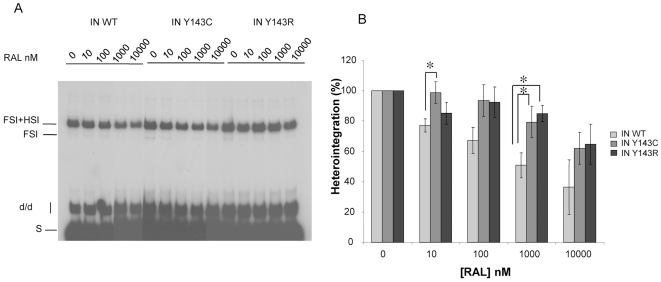
Sensitivity to RAL of IN WT and IN Y143C/R mutants in a concerted integration assay. IN (50 nM) was incubated in presence of donor and acceptor DNA and increasing amounts of RAL. Reaction was then processed as described in material and methods. A typical gel is shown if Figure 6A. Inhibition curves are shown if Figure 6B and are the result of at least three independent experiments. Heterointegration corresponds to densitometry of the FSI and FSI+HIS bands (see products indicated in Figure 6A). A Student Test was used to derive P value. * p value <0.05 considered statistically significant.

The sensitivity to RAL was also measured in a concerted integration assay using uncleaved substrate. In this assay, the sensitivity of wild-type enzyme to RAL was decreased compared to 3′end processing and strand transfer assay (Figure 6). Indeed, 30% of activity was still measured for 10 µM of RAL while this concentration totally inhibited activity in strand transfer and processing assays. The strand transfer function of the mutant recombinant integrases (particularly Y143C) in the concerted integration reaction was not as defective as when the same enzymes were used in the strand transfer reaction with pre-processed DNA. This could be a possible explanation for the disparity observed between both assays. Both mutants were significantly more resistant to RAL than the wild-type enzyme, with 60% of activity (twice as much as the wild type) maintained at a 10 µM concentration of RAL.

## Discussion

RAL was licensed at the end of 2007 as the first HIV-1 INI and is currently prescribed to antiretroviral-experienced and -naïve patients. We set up a prospective study including antiretroviral-experienced patients receiving RAL and an optimized background therapy and found a particular pattern of mutations involving IN mutations Y143C/H/R in patients with virological failure with RAL [14]. In this work, we further investigate the genetic pathways and the dynamics of emergence of the Y143C/R mutations in the HIV-1 integrase gene in three patients failing on RAL-containing regimens, as well as the influence of Y143C/R mutations on IN functions and on the sensitivity of IN to RAL.

When the viruses of these three patients replicated on therapy, selection of the mutations L74M, T97A, Y143C/H/R, N155H, G163R and V201I/L was observed in the population sequences originating from plasma HIV-1 RNA. The profile of the mutations was not the same in all three patients. Moreover, the mutations were not found together in the viral population but appeared sequentially, as shown in Table 2. Interestingly, in all three patients, the N155H mutation appeared at M1 or M3, and then disappeared with a switch to the emergence of the Y143C/H/R mutation detected 3 months later. The Y143C/H/R mutation was associated with T97A in 3 patients, with L74M in 2 patients and with G163R and S230R in one patient each. This co-selection of secondary mutations, particularly T97A, along with the mutations at position 143 are in agreement with other studies [19].

Numerous reverse transcriptase and protease mutations were described in the viral sequence of these patients. In other studies, associations between RT mutations and IN mutations have been reported for patients under HAART. In the three patients studied here, the M154L and V165I mutations, which had been associated with previous antiretroviral experience [11], were not present at baseline. The T206S polymorphism, which was found to be associated with a worse virological response in our original prospective cohort [14], was also absent at baseline. Thus, further research will be needed to specify the determinants of selection of Y143R/C.

Replacement over time of the N155H pattern by the Y143R/C pattern was confirmed by a clonal analysis of the evolution of HIV-1 IN in the three patients, while the Y143H mutation was not confirmed at the clonal level, suggesting that it could be an artifact of the population sequence translation. Clonal analysis suggested that the mutations at positions 143 and 155 were exclusive and were selected independently. Surprisingly, in a recent study on site-directed mutants, the double mutant Y143R + N155H exhibited a selective advantage over other mutants in the presence of RAL [20]. However, different constraints could play a role against the co-selection of these two mutations *in vivo*. Of note, the other major mutation Q148R was transiently detected in one patient in one clone at baseline RAL and at month 6, but did not seem to confer any selective advantage over the other mutants in this patient.

The switch to Y143R/C was clearly associated with a loss of sensitivity to RAL, with fold-changes of 5.87, 28.52 and 51.95 in patients 1, 2 and 3, respectively. EVG sensitivity was less decreased, suggesting that the mutations at position 143 might be less involved in cross-reactivity to EVG.

To observe the effect of mutations Y143C and Y143R on IN activity and RAL sensitivity, the mutated integrases were produced, purified and *in vitro* 3′end processing, strand transfer and concerted integration were assayed according to standard procedures previously used in our laboratory [16], [21]. Sensitivity of the mutants to RAL was measured in processing, strand transfer and concerted integration. Processing activities of the three enzymes were sensitive to RAL. Processing assays were performed in Mn^2+^ and not in Mg^2+^. This will not influence the comparison between wild type and mutant IN. Yet, the drug IC_50_ values might be lower in presence of Mg^2+^, which is the relevant cofactor. The ST activity is more efficiently inhibited when the reaction is performed with the full length substrate (strand transfer observed in 3′end processing assay Figure 4B) versus precleaved substrate (strand transfer assay, Figure 5), as previously observed by Marinello et al [12], suggesting again that it binds to a preformed complex IN-DNA rather than to the enzyme alone. This again shows the specificity of RAL for ST.

The processing activities of mutants Y143C and Y143R were moderately decreased compared to the wild-type enzyme and the mutations more strongly affected the strand transfer activities of both mutants enzymes when using pre-cleaved substrate. The decrease of concerted integration activity that was observed for the mutants is probably due to the defect in strand transfer activity in this assay. Yet, this strand transfer function is not as defective as when the same enzyme was used in the strand transfer reaction with preprocessed DNA. This difference could explain why integrase in concerted integration is relatively insensitive to RAL. Such a difference of inhibition of IN between concerted integration and strand transfer assays was already observed for other inhibitors in our hands (not shown). Furthermore, all the assays were performed under standard procedures set up for optimizing enzyme *in vitro* activities. 3′ processing and strand transfer assay differ from concerted integration in numerous ways including cations requirement. In addition, we previously demonstrated that the IN behaviour on short ODN mimicking the viral ends, as in the case of 3′ processing and strand stransfer *in vitro* reactions, and on longer fragments containing unspecific DNA as in the case of the SupF concerted integration substrate differs. This is due to the need for specific placement of the enzyme on the viral ends on long substrates [17]. Taken together all those molecular differences found between assays could explain the few variations observed for 3′ processing, strand transfer and concerted integration activity in term of inhibitor sensitivity.

How could the selected mutations impair RAL binding while allowing alternative DNA recognition? We observed (data not shown) a lower binding to DNA substrate in the case of mutant Y143R. Residue Y143 in wild-type enzyme has been shown to be part of a highly disordered loop of the catalytic core of IN and to be involved in the binding of the terminal portion of viral DNA ends [22]. Experiments performed with integrase carrying the Y143C mutation [23] showed that it retains single-end strand transfer activity and can crosslink with blunt-end DNA, suggesting that the contact is maintained with the viral DNA during the conformational change between the 3′processing and strand transfer step. This observation is consistent with our finding that the Y143C mutant binds strand transfer substrate. On the other hand, we observed a low binding to DNA substrate in the case of the Y143R mutant which could be responsible for the catalytic defect in this enzyme. Studies of molecular dynamic simulations performed with a dimer of IN [24] demonstrated that in the first IN subunit, the catalytic core does not make direct interactions with viral DNA. However, in the second subunit, the Y143 side chain points completely towards the active site and is favorably orientated for facilitating enzymatic function. Interaction with DNA is probably impaired owing to an unfavorable orientation when Y143 is mutated in R, an orientation which is more favorable when Y143 is mutated in C. This is in agreement with the work of Mouscadet et al [25] who propose that native residues in wild-type enzyme involved in resistance (N155 and Q148) have a clear preference for adenine recognition, while mutations of N155 in H or of Q148 in H, R or K give mutants that favor pyrimidines. It might be that RAL inhibits HIV replication by mimicking adenine. The selected mutations might impair RAL binding while allowing alternative DNA recognition.

Recent data from site-directed mutants showed a high impact of Y143R on replicative capacity and on phenotypic resistance to RAL (Fransen et al, unpublished data). Recently, the impact of the substitutions at residue 143 on the sensitivity to RAL was also investigated [26]. Y143R/C mutations conferred a high resistance to RAL *in vitro* and *in vivo*. The replicative capacity of Y143 mutant virus was dramatically lower than that of the wild-type virus [26]. Despite impairment of IN activities observed *in vitro*, Y143C and Y143R mutants viruses are able to replicate efficiently *in vivo*. So it is possible that protease or reverse transcriptase rescued replication capacity of viruses containing integrase resistance mutations. HIV-1 genes are involved in modulating viral fitness in patients failing on raltegravir-containing regimen [27]. From this point of view, in our *in vitro* study, we analyzed only the IN activities and we could not consider interactions with others HIV-1 genes products. Moreover, the level of resistance was not only linked to the amino acid found in position 143, but was highly modulated by the combination with secondary mutations and also by the back-ground integrase sequence present in patient at baseline [28].

Selection of the Y143R/C mutation was accompanied in all three patients by the T97A mutation. This mutation seems to enhance the resistance to RAL in the presence of Y143C, as suggested by *in vitro* experiments on site-directed mutants (Fransen et al, unpublished data). This could explain the high difference of FC to RAL found between *in vitro* mutant IN with Y143C/R alone, or *ex vivo* with recombinant viruses from patients. Moreover, our cloning analysis showed that some clones could carry T97A in the absence of Y143C/R and other major mutations, suggesting that T97A could be sufficient to code resistance to RAL. Further experiments should focus on the role of T97A by introducing this mutation alone into the integrase gene. Moreover, the association of mutations T97 and Y143 might increase the resistance level to RAL and/or rescue the catalytic defect due to the Y143C/R mutation.

In conclusion, we characterized the selection dynamics of the IN mutations Y143R/C in patients failing on RAL-containing regimens. In all the patients, Y143R/C with secondary mutations replaced the N155H mutation. Y143C/R and N155H seemed to be exclusive *in vivo*. Further research is warranted to investigate whether the impact of Y143C/R on IN functions and on resistance to RAL at the enzymatic level could be modulated by secondary INI resistance mutations.

## Materials and Methods

### Study population and design

The patients originated from a cohort of 51 antiretroviral-experienced patients treated by RAL-containing ART in the setting of an expanded access program in France [14]. The patients were selected from the ANRS Co3 Aquitaine Cohort, a prospective hospital-based cohort of HIV-1 infected patients in south-western France. Written informed consent was obtained for all patients. The Aquitaine Cohort has an Institutional Review Board (IRB) approval from the Bordeaux University IRB.

The RAL dosage was 400 mg twice daily. The co-prescribed antiretroviral drugs were chosen on the basis of a baseline genotypic resistance analysis. The patients were followed up at months 0, 1, 3, 6, 9 and 12 on RAL-containing ART, at which points plasma HIV-1 RNA (CobasTaqman HIV assay, Roche Diagnostics, Basel, Switzerland) and CD4 (+) cell counts were measured. Only patients who had VF (defined as plasma HIV-1 RNA>400 copies/ml after three months on RAL and/or >50 copies/ml after six months on RAL) with selection of mutations at residue 143 were included in this study.

### Amplification and sequencing of the HIV-1 integrase gene

The complete integrase gene was PCR-amplified from plasma samples collected at baseline and at the time of virological failure. Plasma (1 ml) was centrifuged at 19,000 *g* for 1 h at 4°C, and viral RNA was extracted from the pellet using the High Pure Viral RNA Kit (Roche Diagnostics). Ten microliters of RNA were used for RT-PCR (Titan one-Tube RT-PCR kit, Roche Diagnostics) using primers IN12 and IN13. A nested PCR was then performed by using Ampli Taq Gold DNA Polymerase (Applied Biosystems) with primers IN and BH4.

After purification of the amplified DNA (S400 columns, Pharmacia), the integrase gene was sequenced by using two forward (IN1 and IN4542S) and two reverse (IN4764AS and BH4) primers. The complete sequencing procedures and primer sequences are described at www.hivfrenchresistance.org. The sequence analysis was processed on a Beckmann CEQ2000 XL Sequencer and the sequences were aligned on the HXB2 reference sequence by using the SmartGene software. We followed-up the 50 mutations of resistance present at 32 positions: associated with *in vitro* or *in vivo* resistance to HIV-1 integrase inhibitors: H51Y, T66I/A/K, V72I, L74I/A/M, E92Q, T97A, T112I, F121Y, T125K, A128T, E138 K/A/D, G140R/C/H, Y143C/H/R, Q146K/P, S147G, Q148K/R/H, V151I, S153Y/A, M154I, N155S/H, K156N, E157Q, K160D/N, G163 R/K, V165I, V201I, I203M, T206S, S230N/R, V249I, R263K, C280Y. These mutations were previously described by Lataillade et al [29] as associated with *in vitro* or *in vivo* resistance to HIV-1 integrase inhibitors.

### Genotypic resistance analysis

Reverse transcriptase and protease were submitted for genotypic resistance analysis according to the ANRS consensus procedures available at www.hivfrenchresistance.org. The genotypic sensitivity score (GSS) was calculated as the sum of genotypic sensitivities (according to the ANRS genotype-interpretation algorithm, version 17 (http//:www.hivfrenchresistance.org): 0, 0.5 or 1 if resistant, partially susceptible or susceptible, respectively) to the drugs co-prescribed with raltegravir. Enfurvitide prescribed in an enfurvitide-naive patient was considered as an active drug.

### HIV-1 integrase cloning and sequencing

In order to study specific patterns of mutations more extensively, IN gene PCR products amplified from plasma HIV-1 RNA were produced in duplicate for each sample studied and were pooled. The PCR products were then cloned using the pGEM®-T Easy Vector System kit (Promega Corporation) according to the manufacturer's recommendations. The purified clonal fragments were then sequenced (20–50 clones per sample) and linkage between integrase mutations was determined.

### Phenotypic sensitivity to RAL and Elvitegravir (EVG)

#### Viral RNA extraction

Viral RNA was isolated, either starting from 256 µl plasma using the automated QIAamp Virus BioRobot MDx extraction platform (Qiagen), or from 600 µl plasma using the EasyMAG procedure (BioMérieux) according to the manufacturer's instructions.

#### Amplification of the HIV-1 Integrase gene

Starting from viral RNA, cDNA was generated using the Accuscript High Fidelity Reverse Transcriptase (Stratagene) with random hexamers. cDNA synthesis consisted of three steps:

10 min at 25°C, 60 min at 42°C, followed by 15 min at 70°C. Subsequently, the IN gene was amplified by nested PCR using forward primers 5′INoutR1 (positions 4059–4081 in HXB2) and 5′INinF1 (positions 4143–4164 in HXB2) and reverse primers 3′INoutR2 (positions 5241–5262 in HXB2) and 3′INinR1 (positions 5195–5217 in HXB2). Both the outer and inner PCR were performed using the Phusion High-Fidelity PCR Master Mix (Finnzymes). Thermal cycling of both PCRs consisted of a denaturation step at 98°C for 30 sec, 30 cycles of 10 sec at 98°C, 30 sec at 58°C and 30 sec at 72°C, and a final elongation for 10 min at 72°C.

#### Purification and genotyping of the IN amplicons

Amplicons were purified using the QiaQuick PCR purification (Qiagen). IN sequencing reactions were performed using the BigDye Terminator cycle sequencing kit and run on an ABI3730 automated sequencer (Applied Biosystems). Sequence editing and contig assembly were performed using Sequencher v4.1.4 (Gene Codes Corporation) or SeqScape v2.5 (Applied Biosystems) and HXB2 as a reference.

#### Production of replication-competent recombinant viruses

We used an HXB2-based HIV backbone in which the integrase region was deleted (pHXB2-ΔIN) [30], [31]. IN amplicons were then recombined intracellularly in MT4 cells with the pHXB2-ΔIN backbone by Amaxa nucleofection (Amaxa Biosystems) according to the manufacturer's recommendations. The cytopathic effect (CPE) was monitored during the course of infection. When full CPE was reached, recombinant viruses were harvested by centrifugation.

#### Drug susceptibility testing of recombinant viruses

Replication-competent recombinant viruses were titrated and subjected to antiviral testing in MT4-LTR-eGFP cells using RAL and EVG at concentrations from 0.1 nM to 3.6 µM and 0.04 nM to 0.7 µM respectively. After 3 days incubation at 37°C and 5% CO_2_, infection was quantified by means of UV microscopy measuring the HIV Tat-induced eGFP expression. Using the IIIB HIV-1 wild-type virus as a reference, fold change (FC) values were calculated.

### Site-directed mutagenesis and bacterial expression

The integrase region of *pol* gene of pNL4-3 was subcloned in pet21b. Mutagenesis at position 143 was performed using the QuikChange Site-directed Mutagenesis kit (Stratagene) according to the manufacturer's instructions. Plasmids were amplified in DH5α strains.

### IN purification

Standard purification of IN was performed essentially as previously described in our laboratory [15], [17], [21]. Briefly, integrase expression was done in Rosetta bacterial strains. Induction was done for 3 h. The soluble fraction containing the HIV-1 IN was loaded on a Hitrap butyl-sepharose 4B column (1 ml, Pharmacia-LKB), washed with LSC buffer (50 mM HEPES pH 7.6, 0.2 M NaCl, 0.1 M EDTA, 1 mM DTT, 7 mM CHAPS, 10% glycerol) and equilibrated with 5 volumes HSC buffer (50 mM HEPES pH 7.6, 0.2 M NaCl, 1 M ammonium sulfate, 0.1 mM EDTA, 1 mM DTT, 7 mM CHAPS). Proteins were eluted by a decreasing ammonium sulfate gradient (1 to 0 M). Fractions containing IN were pooled and 7 mM CHAPS was added. Pooled fractions were 1/3 diluted with 50 mM HEPES pH 7.6, 0.1 M EDTA, 1 mM DTT, 10% glycerol, 7 mM CHAPS and loaded on a Hitrap Heparine Sepharose CL-4B column (1 ml, Pharmacia-LKB), washed with 5 volumes HS buffer (50 mM HEPES pH 7.6, 1 M NaCl, 0.1 mM EDTA, 1 mM DTT, 10% glycerol, 7 mM CHAPS) and eluted with a linear NaCl gradient (0 to 1 M NaCl). Elution of the three enzymes was observed at the same concentration of NaCl along the purification process. Fractions containing IN activity (3′-end processing and strand transfer assays) were pooled and concentrated or not by ultrafiltration (Centricon Millipore). Purified IN was kept at −80°C. Proteins were analyzed by electrophoresis on a 12% SDS-PAGE. The 3 enzymes have similar purification rate of 99% homogeneity.

### In vitro activities

#### Processing and strand transfer

Standard assays were performed as described previously in 20 mM HEPES pH 7.5, 10 mM DTT, 7.5 mM MnCl_2_, 0.05% NP40 in a total volume of 20 µl [15], [17], [21]. The reaction mixture was incubated at 37°C for 1 h in the presence of IN and radiolabeled oligonucleotides (1 pmol, 50 nM) and the incubation was stopped by adding 10 µl of loading buffer (95% formamide, 20 mM EDTA, 0.05% bromophenol blue) and heating at 90°C for 5 min (standard conditions). The reaction products were analyzed by electrophoresis on 15% polyacrylamide gels with 7 M urea in Tris-borate-EDTA (TBE) pH 7.6 and autoradiographied. The ODN sequences used to perform the processing and strand transfer assays were the following: ODN70: 5′GTGTGGAAAATCTCTAGCAGT3′, ODN71: 5′GTGTGGAAAATCTCTAGCA3′, ODN 72: 5′ACTGCTAGAGATTTTCCACAC3′. To perform the 3′ processing assay, the 5′ radiolabeled ODN 70 hybridized to ODN 72 was used as a substrate while the 5′ radiolabeled ODN 71 hybridized to ODN 72 was used as substrate for the strand transfer reaction.

#### Concerted integration DNA substrates

Standard concerted integration reactions were performed as described previously [16], [17]. Briefly, purified HIV-1 IN (concentrations as indicated in the figures) was pre-incubated with both the 5′-end-labeled donor DNA (10 ng) containing the 3′-unprocessed U3 and U5 LTR sequences and the target DNA plasmid pBSK^+^ (100 ng) at 0°C for 20 min in a total volume of 5 µl. Then the reaction mixture (20 mM HEPES, pH 7.5; 10 mM DTT; 7.5 mM MgCl_2_; 10% DMSO; 8% PEG) was added and the reaction proceeded for 90 min. Incubation was stopped by adding a phenol/isoamyl alcohol/ chloroform mix (24/1/25 v/v/v). The aqueous phase was loaded on a vertical 1% agarose gel in the presence of 1% bromophenol blue and 1 mM EDTA. After separation of the products, the gel was treated with 5% TCA for 20 min, dried and autoradiographied. All IN activities were quantified by scanning of the bands after gel electrophoresis and autoradiography using the Image J software. Both target and donor plasmids were kind gifts from Dr. Karen Moreau (Université Claude Bernard-Lyon I, France). The target corresponds to the plasmid pBSK^+^ (Stratagene, La Jolla, California) carrying the zeocin resistance-encoding gene. The unprocessed donor was generated by cloning a donor containing *Sca*I ends into a pGEM-T vector (Promega) as previously described [16]. The pGEM-T-SupFScaI resulting vector was cleaved by *Sca*I and the substrate fragment was purified.

## Supporting Information

Table S1 Clonal analysis of virological evolution of HIV-1 integrase in three patients failing on raltegravir-containing regimens. Time on therapy: time on raltegravir-containing regimens (Months). Bulk/clones: Bulk when indicated, or number of clones (%).
(0.18 MB DOC)
