# Supplementary material for: The HIV-1 Integrase Mutations Y143C/R Are an Alternative Pathway for Resistance to Raltegravir and Impact the Enzyme Functions
Source: PLoS One. 2010 Apr 26;5(4):e10311. doi: 10.1371/journal.pone.0010311 (PMC2859942; doi:10.1371/journal.pone.0010311)
Supplement: Table S1 — Clonal analysis of virological evolution of HIV-1 integrase in three patients failing on raltegravir-containing regimens. Time on therapy: time on raltegravir-containing regimens (Months). Bulk/clones: Bulk when indicated, or number of clones (%). (0.18 MB DOC) [file pone.0010311.s001.doc]

**Table S1**:

| Patient N° | Time on therapy | Viral population (bulk/clones) | Integrase mutations | | | | | | | | | | | | | | | | | | |
| --- | --- | --- | --- | --- | --- | --- | --- | --- | --- | --- | --- | --- | --- | --- | --- | --- | --- | --- | --- | --- | --- |
|  |  |  | 51 | 72 | 74 | 97 | 138 | 143 | 148 | 151 | 153 | 154 | 155 | 157 | 160 | 163 | 201 | 203 | 230 | 263 | 280 |
| 1 | M0 | Bulk | - | I | - | - | - | **-** | **-** | - | - | - | - | - | - | - | - | M | - | - | - |
|  |  | 1/15 (6.7) | - | I | - | - | - | **-** | **-** | - | - | - | **S** | - | - | - | - | M | - | - | - |
|  |  | 1/15 (6.7) | - | I | - | - | - | **-** | **-** | - | - | V | **-** | - | - | R | - | M | - | - | - |
|  |  | 1/15 (6.7) | - | I | - | - | - | **-** | **-** | - | - | - | **-** | - | - | - | - | M | - | - | R |
|  |  | 12/15 (80) | - | I | - | - | - | **-** | **-** | - | - | - | **-** | - | - | - | - | M | - | - | - |
| 1 | M1 | Bulk | - | I | - | - | - | **-** | **-** | - | - | - | **H/N** | - | - | - | - | M | - | - | - |
|  |  | 5/18 (28) | - | I | - | - | - | **-** | **-** | - | - | - | **H** | - | - | - | - | M | - | - | - |
|  |  | 2/18 (11) | - | I | - | - | - | **-** | **-** | - | - | - | **H** | - | - | R | - | M | - | - | - |
|  |  | 2/18 (11) | - | I | - | - | - | **-** | **-** | - | - | - | - | Q | - | - | - | M | - | - | - |
|  |  | 1/18 (6) | - | I | - | - | - | **-** | **-** | - | - | - | - | - | - | - | - | M | G | - | - |
|  |  | 8/18 (44) | - | I | - | - | - | **-** | **-** | - | - | - | - | - | - | - | - | M | - | - | - |
| 1 | M3 | Bulk | - | I | L/M | A/T | - | **C** | **-** | I/V | - | - | - | - | - | - | - | M | R | - | - |
|  |  | 1/11 (9) | - | I | - | - | - | **C** | **-** | - | - | - | - | - | - | - | - | M | - | - | - |
|  |  | 1/11 (9) | - | I | - | A | - | **C** | **-** | - | - | - | - | - | - | - | - | M | - | - | - |
|  |  | 5/11 (46) | - | I | - | - | - | **C** | **-** | - | - | - | - | - | - | - | - | M | R | - | - |
|  |  | 2/11 (18) | - | I | M | - | - | **C** | **-** | - | - | - | - | - | - | - | - | M | R | - | - |
|  |  | 1/11 (9) | R | I | - | - | - | **C** | **-** | - | - | - | - | - | - | - | - | M | R | - | - |
|  |  | 1/11 (9) | - | I | - | - | - | **-** | **-** | I | - | - | **H** | - | - | - | - | M | R | - | - |

| Patient N° | | Time on therapy | Viral population (bulk/clones) | Integrase mutations | | | | | | | | | | | | | | | | | | |
| --- | --- | --- | --- | --- | --- | --- | --- | --- | --- | --- | --- | --- | --- | --- | --- | --- | --- | --- | --- | --- | --- | --- |
|  |  |  |  | 51 | 72 | 74 | 97 | 138 | 143 | 148 | 151 | 153 | 154 | 155 | 157 | 160 | 163 | 201 | 203 | 230 | 263 | 280 |
| 2 | M0 | | Bulk | - | - | - | - | - | **-** | **-** | I/V | - | - | **-** | - | - | - | - | - | - | - | - |
|  |  | | 1/49 (2) | - | - | - | - | - | **-** | **R** | - | - | - | **-** | - | - | - | - | - | - | - | - |
|  |  | | 22/49 (45) | - | - | - | - | - | **-** | **-** | - | - | - | **-** | - | - | - | - | - | - | - | - |
|  |  | | 1/49 (2) | - | - | - | A | - | **-** | **-** | - | - | - | **-** | - | - | - | - | - | - | - | - |
|  |  | | 3/49 (6) | - | - | - | - | - | **-** | **-** | - | - | - | **-** | - | - | - | - | M | - | - | - |
|  |  | | 1/49 (2) | - | - | - | - | - | **-** | **-** | - | - | - | **-** | - | - | R | - | - | - | - | - |
|  |  | | 1/49 (2) | - | - | - | - | - | **-** | **-** | - | - | - | **-** | - | - | - | - | - | N | - | - |
|  |  | | 20/49 (41) | - | - | - | - | - | **-** | **-** | I | - | - | **-** | - | - | - | - | - | - | - | - |
| 2 | M3 | | Bulk | - | - | - | - | - | **-** | **-** | - | - | - | **H** | - | - | - | - | - | - | - | - |
|  |  | | 1/45 (2) | - | - | - | - | - | **-** | **-** | - | - | - | **R** | - | - | - | - | - | - | - | - |
|  |  | | 29/45 (65) | - | - | - | - | - | **-** | **-** | - | - | - | **H** | - | - | - | - | - | - | - | - |
|  |  | | 1/45 (2) | - | - | - | - | - | **-** | **-** | I | - | - | **H** | - | - | - | - | - | - | - | - |
|  |  | | 1/45 (2) | - | - | - | - | - | **-** | **-** | I | - | - | **H** | - | - | - | A | - | - | - | - |
|  |  | | 1/45 (2) | R | - | - | - | - | **-** | **-** | - | - | - | **H** | - | - | - | - | - | - | - | - |
|  |  | | 1/45 (2) | - | - | - | - | - | **-** | **-** | - | - | T | **H** | - | - | - | - | - | - | - | - |
|  |  | | 2/45 (5) | - | - | - | - | - | **-** | **-** | - | - | - | **H** | - | - | R | - | - | - | - | - |
|  |  | | 2/45 (5) | - | - | - | - | - | **-** | **-** | - | - | - | **H** | - | - | - | - | M | - | - | - |
|  |  | | 4/45 (9) | **-** | - | - | A | - | **R** | **-** | - | - | - | **-** | - | - | R | - | - | - | - | - |
|  |  | | 1/45 (2) | - | - | - | - | - | **-** | **-** | - | - | - | **-** | - | - | - | - | - | - | - | - |
|  |  | | 1/45 (2) | - | - | - | - | - | **-** | **-** | - | - | - | **-** | - | R | - | - | - | - | - | - |
|  |  | | 1/45 (2) | - | - | - | - | - | **-** | **-** | - | - | - | **-** | - | - | R | - | - | - | - | - |
| 2 | M6 | | Bulk | - | - | - | A/T | - | **CHRY** | **-** | - | - | - | **-** | - | - | G/R | - | - | - | - | - |
|  |  | | 2/47 (4.5) | - | - | - | A | - | **-** | **R** | - | - | - | **-** | - | - | - | - | - | - | - | - |
|  |  | | 3/47 (6.5) | - | - | - | A | - | **R** | **-** | - | - | - | **-** | - | - | - | - | - | - | - | - |
|  |  | | 3/47 (6.5) | - | - | - | - | - | **R** | **-** | - | - | - | **-** | - | - | R | - | - | - | - | - |
|  |  | | 21/47 (45) | - | - | - | A | - | **R** | **-** | - | - | - | **-** | - | - | R | - | - | - | - | - |
|  |  | | 2/47 (4.5) | - | - | P | A | - | **R** | **-** | - | - | - | **-** | - | - | R | - | - | - | - | - |
|  |  | | 1/47 (2) | - | - | - | A | - | **R** | **-** | - | - | - | **-** | K | - | R | - | - | - | - | - |
|  |  | | 1/47 (2) | - | - | - | A | - | **R** | **-** | - | - | - | **-** | - | - | R | - | - | - | G | - |
|  |  | | 1/47 (2) | - | - | - | - | - | **-** | **-** | - | F | - | **-** | - | - | - | - | - | - | - | - |
|  |  | | 1/47 (2) | **-** | - | - | A | - | **-** | **-** | - | - | - | **-** | - | - | R | - | - | - | - | - |
|  |  | | 7/47 (15) | - | - | - | - | - | **-** | **-** | - | - | - | **-** | - | - | - | - | - | - | - | - |
|  |  | | 2/47 (4) | - | - | - | - | - | **-** | **-** | - | - | - | **-** | - | - | R | - | - | - | - | - |
|  |  | | 1/47 (2) | - | - | - | A | - | **-** | **-** | - | - | - | **-** | - | - | R | - | M | - | - | - |
|  |  | | 1/47 (2) | - | - | - | A | - | **-** | **-** | - | - | - | **-** | - | - | - | - | - | - | - | - |
|  |  | | 1/47 (2) | - | - | - | A | G | **-** | **-** | - | - | - | **-** | - | - | - | - | - | - | - | - |

| Patient N° | Time on therapy | Viral population (bulk/clones) | Integrase mutations | | | | | | | | | | | | | | | | | | |
| --- | --- | --- | --- | --- | --- | --- | --- | --- | --- | --- | --- | --- | --- | --- | --- | --- | --- | --- | --- | --- | --- |
|  |  |  | 51 | 72 | 74 | 97 | 138 | 143 | 148 | 151 | 153 | 154 | 155 | 157 | 160 | 163 | 201 | 203 | 230 | 263 | 280 |
| 3 | M0 | Bulk | - | I | - | - | - | - | - | - | - | - | - | - | - | - | - | - | - | - | - |
|  |  | 1/16 (6) | - | I | - | A | - | - | - | - | - | - | - | - | - | - | - | - | - | - | - |
|  |  | 2/16 (13) | - | I | - | - | - | - | - | I | - | - | - | - | - | - | - | - | - | - | - |
|  |  | 1/16 (6) | - | I | - | - | - | - | - | - | - | - | - | - | R | - | - | - | - | - | - |
|  |  | 12/16 (75) | - | I | - | - | - | - | - | - | - | - | - | - | - | - | - | - | - | - | - |
| 3 | M3 | Bulk | - | I | - | - | - | - | - | - | - | - | **H/N** | - | K/N | - | - | - | - | - | - |
|  |  | 1/18 (6) | - | I | - | - | - | - | - | - | - | - | **-** | - | R | - | - | - | - | - | - |
|  |  | 11/18 (60) | - | I | - | - | - | - | - | - | - | - | **H** | - | - | - | - | - | - | - | - |
|  |  | 1/18 (6) | **-** | I | - | - | - | - | - | I | - | - | **H** | - | - | - | - | - | - | - | - |
|  |  | 1/18 (6) | **-** | I | - | - | - | - | - | - | - | - | **H** | - | - | - | I | - | - | - | - |
|  |  | 4/18 (22) | - | I | - | - | - | - | - | - | - | - | - | - | - | - | - | - | - | - | - |
| 3 | M6 | Bulk | - | I | - | A/T | - | **CHRY** | - | - | - | - | - | - | - | - | - | - | - | - | - |
|  |  | 7/23 (30) | - | I | - | - | - | **R** | - | - | - | - | - | - | - | - | - | - | - | - | - |
|  |  | 1/23 (4) | **-** | I | - | - | - | **R** | - | - | - | I | - | - | - | - | - | - | - | - | - |
|  |  | 1/23 (4) | - | I | - | - | - | **R** | - | - | - | - | - | - | - | - | - | - | - | - | - |
|  |  | 2/23 (9) | - | I | - | A | - | **R** | - | - | - | - | - | - | - | - | - | - | - | - | - |
|  |  | 8/23 (36) | - | I | - | A | - | - | - | - | - | - | - | - | - | - | - | - | - | - | - |
|  |  | 1/23 (4) | - | I | - | A | - | - | - | - | - | - | - | K | - | - | - | - | - | - | - |
|  |  | 1/23 (4) | - | V | - | A | - | - | - | - | - | - | - | - | - | - | - | - | - | - | - |
|  |  | 2/23 (9) | - | I | - | - | - | - | - | - | - | - | - | - | - | - | - | - | - | - | - |
